# Microbubbles Stabilized by Protein Shell: From Pioneering Ultrasound Contrast Agents to Advanced Theranostic Systems

**DOI:** 10.3390/pharmaceutics14061236

**Published:** 2022-06-10

**Authors:** Polina G. Rudakovskaya, Roman A. Barmin, Pavel S. Kuzmin, Elena P. Fedotkina, Alexander N. Sencha, Dmitry A. Gorin

**Affiliations:** 1 Center for Photonic Science and Engineering, Skolkovo Institute of Science and Technology, Nobel Str. 3, 121205 Moscow, Russia; roman.barmin@skoltech.ru; 2 Institute of Materials for Modern Energy and Nanotechnology, Dmitry Mendeleev University of Chemical Technology of Russia, Miusskaya Sq. 9, 125047 Moscow, Russia; pavelkuzbmin@gmail.com; 3 Research Center for Obstetrics, Gynecology and Perinatology, Ministry of Healthcare of the Russian Federation, Akademika Oparina Str. 4, 117198 Moscow, Russia; epf@yandex.ru (E.P.F.); asencha@yandex.ru (A.N.S.)

**Keywords:** ultrasound, microbubbles, contrast agents, proteins, albumin, lysozyme, oleosin, drug delivery, theranostics

## Abstract

Ultrasound is a widely-used imaging modality in clinics as a low-cost, non-invasive, non-radiative procedure allowing therapists faster decision-making. Microbubbles have been used as ultrasound contrast agents for decades, while recent attention has been attracted to consider them as stimuli-responsive drug delivery systems. Pioneering microbubbles were Albunex with a protein shell composed of human serum albumin, which entered clinical practice in 1993. However, current research expanded the set of proteins for a microbubble shell beyond albumin and applications of protein microbubbles beyond ultrasound imaging. Hence, this review summarizes all-known protein microbubbles over decades with a critical evaluation of formulations and applications to optimize the safety (low toxicity and high biocompatibility) as well as imaging efficiency. We provide a comprehensive overview of (1) proteins involved in microbubble formulation, (2) peculiarities of preparation of protein stabilized microbubbles with consideration of large-scale production, (3) key chemical factors of stabilization and functionalization of protein-shelled microbubbles, and (4) biomedical applications beyond ultrasound imaging (multimodal imaging, drug/gene delivery with attention to anticancer treatment, antibacterial activity, biosensing). Presented critical evaluation of the current state-of-the-art for protein microbubbles should focus the field on relevant strategies in microbubble formulation and application for short-term clinical translation. Thus, a protein bubble-based platform is very perspective for theranostic application in clinics.

## 1. Introduction

**Figure 1:**
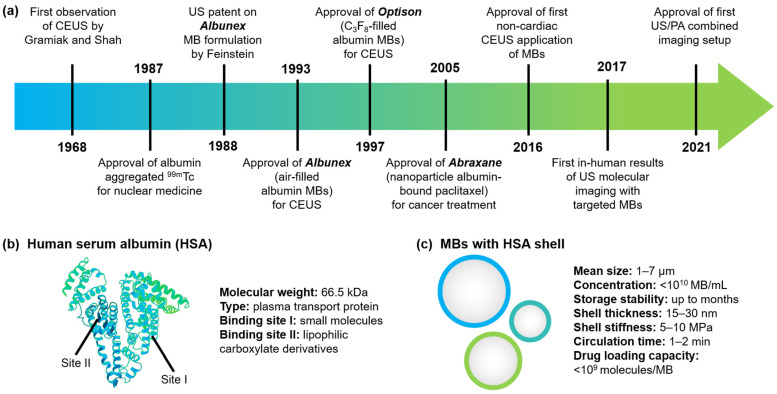
Microbubbles with albumin shell for ultrasound imaging. (**a**) Timeline of key advances in the field of ultrasound (US) contrast agents and albumin-based formulations development; key characteristics of (**b**) human serum albumin (HSA) and (**c**) microbubbles with an HSA shell. Abbreviations: US, ultrasound; CEUS, contrast-enhanced ultrasound; MBs, microbubbles; PA, photoacoustic; HSA, human serum albumin.

Ultrasound (US) imaging is a workhorse in clinical diagnostics routine, as it is non-invasive, low-cost, and requires no ionizing radiation [1,2]. US outperforms the number of magnetic resonance imaging (MRI) and computed tomography (CT) examinations by 2–3 times [3,4,5]. US relies on the piezoelectric effect when a device stimulated by an electric current emits and transmits ultrasound pulses and receives reflected echoes from organs and tissues to construct the image [6]. Poor contrast image quality can limit US usability for pathology diagnosis applications, such as hypervascular malignancies, or breast, liver, and renal masses [7,8]. The administration of contrast agents, initially demonstrated by Gramiak and Shah in 1968, can provide echogenicity several orders of magnitude higher than solid particles of comparable size [9,10,11,12]. Over the years, gas-filled microbubbles (MBs) have become the most popular US contrast agents with the ability to behave as a non-linear oscillator and increase the detected signal intensity up to 1000 times. A brief timeline of the development of US imaging with contrast agents together with the development of albumin-based formulations is presented in Figure 1a.

MBs are colloidal systems with a mean diameter of 1–7 µm, acting as real blood pool agents. Beyond imaging applications, MBs are actively investigated as drug delivery systems due to their shell drug loading capacities and gaseous core US stimuli-responsiveness [10,13,14,15,16]. An MB shell is mainly stabilized with lipids, proteins, or polymers [17,18,19,20,21,22,23,24]. Shell composition primarily affects MB performance regarding storage stability, circulation time, and stimuli response [25,26,27,28,29]. While lipid-based soft shell MBs are preferred for US imaging due to their optimal MB oscillation and resulting contrast profile; still, the gas can intensively diffuse, shortening MB stability. In contrast, polymeric hard shell MBs are preferred for drug delivery as their thicker shell can be loaded with higher amounts of drug molecules and prolongs MB stability, while a thicker shell can reduce contrast [13,17,22].

Protein MBs are a compromise solution with moderate properties between the soft lipid MB oscillation profile and the hard polymer MB drug loading capabilities [22,30,31,32,33] Albumin is one of the most prominent proteins for pharmaceutics [34,35,36]. The most abundant circulating protein in plasma, albumin serves as a versatile carrier for drug delivery and prolongs the active profile of fast-clearance drugs (Figure 1b). “Heart-shaped” protein, human serum albumin (HSA), provides two binding sites as pockets for small molecules, preferably aromatic dyes (by binding site 1) and lipophilic carboxylate derivatives (by binding site 2) [37,38,39,40]. In the past two decades, several HSA-based formulations have been approved by the Food and Drug Administration (FDA) for treatment [34,41].

Pioneering works of MB shell stabilization with albumin by Feinstein and Keller led to the regulatory approval of Albunex (Molecular Biosystems Inc., San Diego, CA, USA) in the USA in 1993 [42,43,44]. Albunex became the first commercially available left-heart US contrast agent in the country with the formulation of sonicated human albumin and air [44]. Such an agent has revolutionized diagnostic US potential; however, MBs were pressure-sensitive, providing only a short-left ventricle contrast duration. Improved formulation of Albunex with a perfluorocarbons (C_3_F_8_) gas core instead of air demonstrated prolonged stability, reached approval in the USA in 1997 and is available as Optison (GE Healthcare AS, Oslo, Norway) [45,46,47,48,49]. Optison became the first US contrast agent using a gas other than air, opening the room for the approval of MBs loaded with perfluorocarbons as lipid-shelled Sonazoid (GE Healthcare AS, Oslo, Norway) and SonoVue (Bracco Suisse SA, Geneva, Switzerland) [50,51,52]. Recent works explore albumin-shelled MBs as drug delivery devices, exploring their applications beyond US imaging and aiming for short-term translation [20,53,54,55]. Key characteristics of HAS-shelled MBs are presented in Figure 1c.

Nowadays, ultrasound expands applications beyond imaging, especially with the MB introduction (Figure 1a). In 2016, MBs gained the US Food and Drug Administration approval for non-cardiac contrast [56]. First-in-human results of US molecular imaging with targeted agents were demonstrated in 2017 [57,58]. Targeted agent formulations entered clinical trials for tumor detection and liver lesion characterization [56,59]. In 2021, the combined ultrasound/photoacoustic imaging setup, the Imagio Breast Imaging System (Seno Medical Instruments, Inc., San Antonio, TX, USA), was approved for commercialization by the FDA, raising the question of smart multimodal/multifunctional agents development for advanced imaging [60,61] Thus, therapeutic-aimed protein MBs act as an optimal candidate for short-term translation [20].

To boost the development of MBs with a protein shell for theranostics, it is crucial to summarize all efforts within the past two decades on (1) formulation of protein MBs, (2) advanced functionalization of the protein shell, and (3) application-driven implementation in biomedicine. Therefore, this review aims to summarize and critically evaluate known examples of protein MBs to enhance their applications in clinical practice.

## 2. Proteins Involved in MB Shell Stabilization

**Figure 2:**
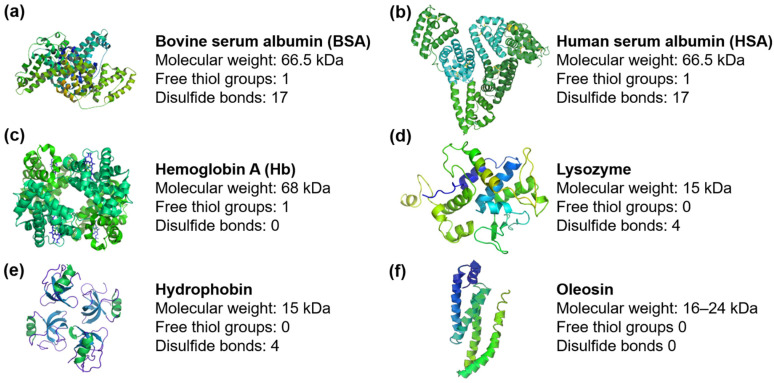
Proteins involved in MB shell stabilization: (**a**) bovine serum albumin (BSA), (**b**) human serum albumin (HSA), (**c**) hemoglobin, (**d**) lysozyme, (**e**) hydrophobin, (**f**) oleosin. For all proteins, characteristics such as molecular weight, numbers of free thiol groups, and disulfide bonds involved in MB formation and further functionalization, are provided.

MB formation is enabled by lowering the surface tension of solutions at the gas-liquid interface with the introduction of surfactants [18,62,63,64]. Hence, various surfactants are used as the basis for MB fabrication. One of the optimal MB shell components is proteins [65,66]. Proteins are biocompatible polymers; natural proteins contain all-natural amino acids. Their presence provides protein amphiphilicity and a wide range of established functionalization routes. An important and insufficiently disclosed area at the moment is synthetic biopolymers based on oligopeptides for MB fabrication. At the moment, the main natural proteins used for MB fabrication are (Figure 2): bovine and human serum albumins (BSA and HSA) [43,44,66,67,68,69,70,71,72,73,74,75,76,77,78,79,80,81,82,83,84], hemoglobin [85], lysozyme [86,87,88,89,90,91,92,93], hydrophobin [94], and oleosin [95,96].

The formation of MBs with protein shells occurs not only due to physical but also chemical processes. However, it remains a challenge to figure out the primary and leading process in MB shell stabilization. The formation, growth, and collapse of MBs can occur during intense local heating, which causes a change in the secondary and tertiary structures of the protein and can also cause high-energy chemical reactions [85]. Moreover, researchers emphasize the role of the radicals formed during the reaction [97]. A study was carried out to identify which particular radical is important for obtaining stable MBs [98]: chemical traps for various types of radicals were added to the MB formation process, and the effect was monitored for the possibility of fabrication, stability, and concentration of agents. The effect of catalase, which decomposes hydrogen peroxide (H_2_O_2_), and superoxide dismutase, which decomposes superoxide (HO_2_) [99], were tested. Superoxide dismutase blocked the formation of MBs, which presumably indicates the participation of superoxide in the reaction of MB formation. Furthermore, it is known that superoxide dismutase easily oxidizes cysteine residues present in many natural proteins used for synthesis [100].

Several studies [85,86,90,91,92,93,98] indicated that the formation and destruction of disulfide bonds play an important role in MB formation with a protein shell. Thus, in [98], hemoglobin and myoglobin were compared: the main difference between proteins is the presence of cysteines in the structure. The latter does not have thiol groups and, as shown, did not form MBs. The addition of cross-linking reagents (i.e., dithiothreitol, DTT) also has been already shown to stabilize the MB structure [66,90,91,92,93]. However, the addition of dithioerythritol, DTE, which destroys disulfide bonds or the process of alkylation of the thio-group, leads to MB destruction.

It should be noted that the energy during the reaction of MB formation is sufficient to destroy the labile existing S-S disulfide bond and form a new one. Furthermore, ref. [67] revealed an increase in the stability and concentration of agents when the protein was pretreated with the Traut’s Reagent (2-iminothiolane), which provides an additional amount of thio-groups by conversion of free amino groups into thio- groups. However, many studies on protein MB formation have shown a dependence not only on the number of thio-groups but also on other possible factors (i.e., the protein structure). For example, the non-universality of the “thiol” substantiation of the MB formation mechanism can be illustrated with MBs stabilized with sulfur-free proteins, such as streptavidin [101] or oleosin [95,96]. Hence, the procedure of MB formation is selected individually for each protein, with its molecular weight, protein structure, and the number of groups that can be involved in the reaction.

All-natural proteins used have the advantage of low toxicity and high biocompatibility. In this aspect, HSA is an ideal candidate for the clinical translation of protein-based US contrast agents. In most studies, due to the high cost of HSA, its less expensive analog, BSA, is used. Its amino acid sequence is 75% identical to the molecular structure of HSA; HSA and BSA have the same molecular weight and behave similarly during MB fabrication [66,102,103]. During numerous experiments with albumin-based MBs, hypotheses about changes in the globular structure of proteins were expressed. The protein MB shell may be formed by close packing of reagents, where hydrophobic fragments of neighboring proteins are tightly packed, and the former intramolecular disulfide bonds are rearranged into intermolecular disulfide bridges. Unfortunately, there is no explicit confirmation of the protein structure inside the shell. However, in [79], it was reported that the MB destruction time correlates with the decrease in the percentage of α-helices during synthesis, which can be considered as an indirect argument in favor of stabilization by unfolded protein molecules.

Proteins with high molecular weight, such as hemoglobin and streptavidin, are used much less frequently compared to albumins. Proteins with significantly lower molecular weights raised interest. Studies on comparing low and high molecular weight proteins (i.e., lysozymes and albumins) did not reveal significant differences in MB properties. However, conditions for MB fabrication were selected individually for each case [66,93,104]. From this perspective, lysozyme demonstrated its potential for protein MB formation due to its unique enzymatic, antibacterial activity [90,91,92,104]. However, the introduction of the universal protocol for the fabrication of protein MBs with desired properties is still yet to come.

## 3. Fabrication of MBs with Protein Shell

**Figure 3:**
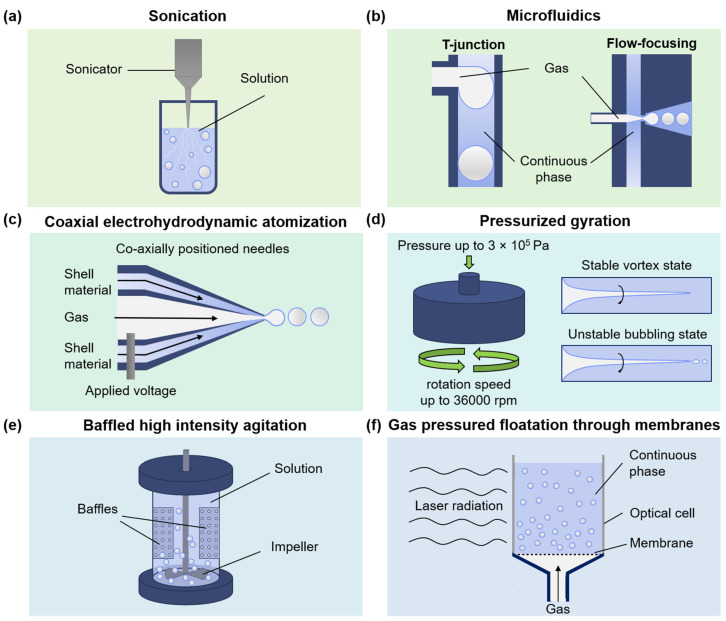
Strategies for protein MBs fabrication. (**a**) Sonication, (**b**) flow-focusing and T-junction microfluidics, (**c**) coaxial electrohydrodynamic atomization (CEHDA), (**d**) pressurized gyration, (**e**) baffled high-intensity agitation (BHIA), and (**f**) gas-pressured floatation through membranes.

MB properties relevant to clinical translation (mean diameter, monodispersity, stability) are tied to the initial fabrication procedure; hence, this section summarizes known approaches to produce MBs with protein shells. Methods include a traditionally referred sonication procedure, a recently explored microfluidic approach, and four techniques designed to increase the volume of produced MBs. Schemes of fabrication routes are summarized in Figure 3.

During the sonication procedure, MBs are primarily formed due to the propagation of high-intensity US waves through a liquid resulting in a cavitation process (Figure 3a) [19,78,97,105,106,107,108,109,110,111]. Typically, the tip of the sonotrode is placed at the gas-liquid interface for MBs fabrication, similar to the formation of micelles, where the tip is placed at the water-oil interface. Sonication is the leading MB production method since chemical laboratories usually have the required setup. However, the method’s main disadvantage is MB polydispersity, affecting their US performance [66]. Monodisperse-sized MBs can demonstrate reduced echo-to-echo decorrelation [112] and enhanced drug delivery properties [113].

Microfluidics aimed to overcome the limitation of polydispersity in MB fabrication. Another advantage of microfluidics is the precise control over reagents involved in MB fabrication. In flow-focusing devices, inner channels with a gaseous phase and outer channels with a continuous phase are merged into a small orifice, leading to MB formation. In T-junction devices, a continuous phase channel is placed perpendicular to a gas phase channel; thus, when gas penetrates the continuous phase under required pressure and flow velocity, local instability at the gas-liquid interface results in MB formation (Figure 3b) [10,114,115,116,117]. However, the scalability of MB production with microfluidics remains the main current limitation.

Coaxial electrohydrodynamic atomization (CEHDA) involves two co-flowing media subjected to a high voltage under ambient conditions to generate coaxial jetting, acting as a compromise between sonication and microfluidics with the advantage of high and scalable MB yield (Figure 3c) [118,119,120]. However, protein MBs are typically large-sized (40–800 µm) compared to lipid MBs [120] produced by CEHDA. Additionally, the high voltage (of 12.8 kV) applied in CEHDA might be a limitation for translation [87].

Pressurized gyration is based on centrifugal spinning and solution blowing to form nanofibers in large quantities and results in the parallel formation of many nanofibers with regular morphology (Figure 3d) [87]. Several articles considered lysozyme-based MB fabrication for biosensing and antibacterial activity [87,121]. Still, the narrowest possible mean size of MBs was only 37 μm, and additional work on expanding pressurized gyration’s speed and pressure regimes are required to meet the size criteria of biocompatible agents.

Baffled high-intensity agitation (BHIA) cells were recently involved in MB formation. The hydrodynamic cavitation of fluid near the impeller and baffles zone at the expense of dissipating more turbulent energy leads to MB formation with sizes smaller than 10 μm, as recently shown for BSA MBs (Figure 3e) with different gases loaded in the core [70]. O_2_- and N_2_-filled MBs were stable for 16 h with mean sizes of 3.7 ± 3.3 μm and 4.4 ± 2.2 μm, respectively. Interestingly, a BSA solution volume of 350 mL was used, scaling the production capabilities of protein-shelled MBs. Further work should be focused on producing narrow-sized MBs by tuning the setup or introducing size separation after MB formation.

Gas pressured floatation through membranes was also implemented for MB formation (Figure 3f): uniform-sized MBs grow at the pore openings of the inner membrane surface, and when the MB volume reaches a specific size, it detaches from the inner surface of the membrane [122,123]. Additionally, further limiting MB size to a 1–7 μm range is required.

However, only a few methods can produce protein MBs with the sizes required for medical applications (<7 μm) and have already gained numerous investigations: sonication (the first historically described method) and microfluidics (recently developed method for fine-tuning MB properties such as monodispersity, control over the MB yield and precise direct functionalization routes). Therefore, we will focus attention on sonication and microfluidic-based methods of MB fabrication to discuss recent advances in the field.

### 3.1. Sonication

A broad set of parameters of the sonication procedure of MB fabrication can be varied: preheating temperature of the initial solution, sonication time, tip location (at the gas-liquid interface or slightly deeper in the solution), US power, frequency, and time of storage for MB stabilization (Figure 3a) [10,19,66]. Since formed MBs are polydisperse in size and formed in solutions with excess reagents, size isolation and MB purification are needed. Upadhyay and Dalvi thoroughly described protein MBs fabricated by sonication in [66].

For example, BSA or HSA solutions can be heated up to 60 °C reaching 80 ± 5 °C during a procedure. The preheating of the initial solution can be used to change protein structure or lead to its denaturation. Moreover, the increasing temperature can lower the solution’s surface tension and assist in forming MB with narrow size distribution [19,21]. The sonication procedure can last from 15 s to 5 min with the sonotrode power of 20–240 W and a US frequency of 20 kHz [66].

The MB size distribution can be tuned by sonication power and time of exposure during sonication [30,112]. Moreover, post-sonication promises size tuning after MB formation, as demonstrated for lysozyme MBs [93]. Narrow size distribution with post-sonication can be achieved by (i) lowering US frequency or (ii) increasing acoustic power at a fixed acoustic power.

The ease of sonication technique combined with a set of predefined parameters to control during the MB synthesis resulted in the widespread use of the method for MB fabrication. Still, MB size polydispersity remains a challenge. Therefore, a microfluidic-based approach was described recently to solve this issue.

### 3.2. Microfluidics

Ideal microfluidic-based fabrication may allow producing (i) MBs with predefined diameter in the range of 1–7 µm and small size polydispersity, which is relevant to advance US contrast properties, (ii) MBs with the controllable concentration needed for the procedure, which can be a crucial advantage for clinical translation of the technology [10,114,115,116,117]. With two types of microfluidic devices highlighted in Figure 3b, all known examples of protein MB microfluidic-assisted MB fabrication are listed in Table 1.

The pioneering work on protein MB fabrication with microfluidics was reported in 2013 by Chen et al.: the flow-focusing device produced agents with BSA or blood plasma shell [124]. Plain protein MBs tended to coalesce rapidly; hence, surfactants (such as dextrose) tuned MB storage stability. The authors demonstrated a step toward MB fabrication with patient blood material; however, the mean diameter was greater than the optimal range of 1–7 µm [124]. Later, the approach that combined MB fabrication and direct administration into a mouse tail vein was performed by Dhanaliwala et al. [126]. Moreover, they demonstrated sonothrombolysis in vitro enhancement when a device was placed in situ adjacent to the clot [127].

Another strategy was proposed by Angilè et al., combining Oleosin with nonionic triblock copolymers poloxamers (i.e., Pluronic F68) [125]. While pure oleosin MBs were larger than 10 µm, the introduction of poloxamers tuned the diameter to 4 µm by lowering the surface tension of initial solutions. Next, tailoring of US response was performed by variation of amphiphilic copolymers in the MB shell: the introduction of longer hydrophilic domains of poloxamers allowed to increase MB shell stiffness [95]. Produced MBs had a diameter of 2–4 µm, were stable over two weeks, and were comparable to commercially available US contrast agents. Later, they produced the bimodal US and photoacoustic agent. Simple electrostatic interactions between oleosin and methylene blue successfully functionalized MBs directly within the microfluidic chamber [96].

Compared to flow-focusing devices, T-junction microfluidic devices produced albumin-shelled MBs with relatively larger diameters of 80–550 µm [79,128,129]. Therefore, flow-focusing devices can produce protein MBs with clinically-relevant properties of size, MB yield, and storage stability.

Therefore, the ideal procedure for protein MB fabrication should offer (i) narrow MB size distribution within the range of 1–7 mm combined with a high MB yield and (ii) demonstrate scalability for the industry implementation combined with ease of implementation. The sonication procedure demonstrates a lack of size monodispersity, microfluidics in the current state is a hardly scalable route for MB production at an industrial scale. However, one of the promising solutions can be direct MB fabrication within a hospital in amounts needed for the department needs, where sonication, microfluidics, and BHIA could be adapted. Moreover, precise attention to the interface phenomena during MB fabrication (i.e., surface tension) could optimize fabrication strategies.

## 4. Chemical Routes for Stabilization and Functionalization of MBs with Protein Shell

**Figure 4:**
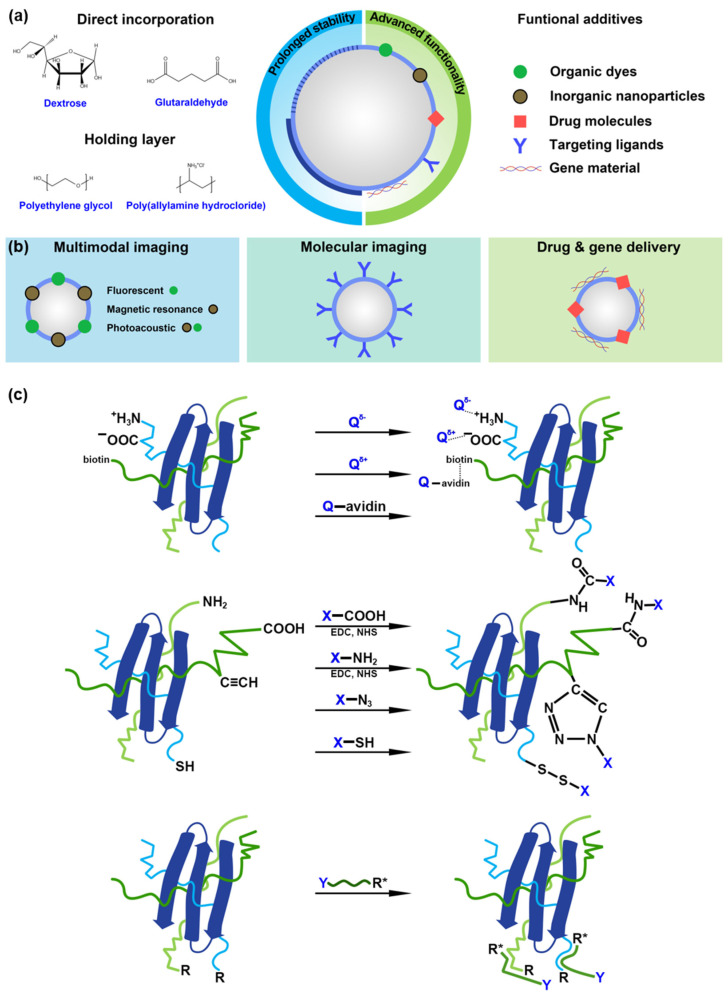
Chemical routes for stabilization and functionalization of MBs with protein shell. (**a**) Prolonged stability of protein MBs can be achieved with direct incorporation of stabilizers (i.e., dextrose or glutaraldehyde) or with the implementation of a “holding” layer of poly(ethylene glycol) or poly(allylamine hydrochloride), while advanced functionalization offers the opportunity to incorporate a broad set of functional additives; (**b**) The proper choice of functional additives results in MB applications for multimodal imaging (where gaseous core provided US imaging, while dyes or nanoparticles provided additional fluorescent/magnetic resonance/photoacoustic modality), molecular imaging (due to targeting ligands), and drug/gene delivery (with functional payloads); (**c**) Schematic representation of chemical routes for protein MB functionalization with non-covalent (electrostatic and “key-lock” with biotin-avidin pair) or covalent interactions (primarily by carbodiimide synthesis or with the introduction of additional crosslinking reagents), as well as hydrophobic interactions.

Protein-based MB functionalization is carried out in two directions: (1) to prolong agent stability (i.e., storage stability, circulation time) and (2) to achieve multifunctionality for applications beyond US imaging. Incorporation of additives into MB shell (noble metal nanoparticles, polymeric nanoparticles, nucleic acids, functional dyes, proteins, and antibodies) expands MB applications to fields of photoacoustic imaging, (targeted) drug/gene delivery, chemo- and photodynamic therapy, antibacterial activity, and even biosensing (Figure 4a).

### 4.1. Prolonged Stability

Two approaches are generally used to achieve prolonged MB stability (Figure 4a). In the first case, active molecules are added during the MB fabrication procedure, contributing to the additional chemical crosslinking of proteins in the MB shell. Several works demonstrated that the addition of reagents having both hydrophobic and hydrophilic fragments in their structure contributes to a denser packing of the MB shell and, consequently, increased stability. For example, Upadhyay et al. [73] produced BSA MBs introducing caprylic acid and N-acetyl-DL-tryptophan in various ratios. Spectroscopic analyses of fluorescence and circular dichroism demonstrated the influence of additives and synthesis conditions on the secondary and tertiary structure of the protein. The use of tryptophan in the MB formulation contributes to the enhanced deployment of BSA molecules, resulting in MBs with a shelf life of up to 8 months at 4 °C, which is comparable to the shell life of polymeric MBs [73,130]. Prolonged stability can be described by forming intermolecular disulfide bonds in the MB shell. In the works [86,90,93,101], a method involved DL-dithiothreitol or β-mercaptoethanol to reduce disulfide bonds and form free thio- groups for later intermolecular coupling. The number of thio- groups can also be increased with the Traut’s reagent [67]: the amino groups included in the protein structure (lysine and arginine) interact with the reagent resulting in the formation of active thio- groups. Another approach involved the covalent crosslinking of proteins with glutaraldehyde as a crosslinking agent [80,109]. The use of such reagents increases stability and circulation time. Additionally, the narrow size distribution of MBs can be achieved.

In another case, the stability is increased by adding a reagent as an additional MB shell layer or as an intermolecular “holding” reagent. In several works, carbohydrates (dextrose and analogs) were involved in protein MB stabilization, a strategy known as “PESDA” [73,74,78,111,124]. Here stabilization occurs due to electrostatic interactions; similar interactions occur with introducing glycerol and ethylene glycols [124,131]. In [74], the strategy of the covalent introduction of polyethylene glycol by carbodiimide binding to the carboxyl group of the protein resulted in desired stability properties of protein MBs. Similarly, in [86], MBs were covalently modified using pre-oxidized dextran followed by the interaction of the aldehyde group with the amino group of the protein.

Several works considered the implementation of Layer-by-Layer assembly on the protein MBs (Figure 4a) [75,77,109,132,133]: unfortunately, this strategy tended to decrease MB concentration and even stability with the addition of each layer. Only one to two layers were sufficient to balance prolonged stability and the further ability for functionalization (which is also possible directly using amino and carboxy groups in the protein).

### 4.2. Advanced Functionality

Two types of interactions can be distinguished for introducing functional groups onto protein MBs: non-covalent (key-lock, electrostatic, or hydrophobic interactions) and covalent (Figure 4b).

The key-lock interactions are widely known by the biotin-avidin (or biotin-streptavidin) interactions. Either biotin fragments [82] or protein [71] are typically introduced into the MB structure, and then efficient coupling is carried out on the MB interface. This approach is directly used for the introduction of antibodies. However, electrostatic binding is the most widespread due to ease of implementation and the presence of positively and negatively charged groups in the protein structure. The elimination of the functional group is the most effective route due to the lack of loss of functional properties. This approach was demonstrated for the introduction of active molecules (i.e., ascorbic acid [134], cysteine [135], nucleic acids or synthetic oligonucleotides [81,131,136], antibodies, enzymes and other peptides [68,73,74,86]), and gold nanoparticles with different morphology [19,21,71,137].

Covalent binding is typically carried out with carbodiimide synthesis or by introducing additional crosslinking reagents. Attachment of antibodies [89] and nanoparticles containing nucleic acids [80] was implemented by maleimide and glutaraldehyde, respectively. Liu et al. [83,84] demonstrated the option of click reaction for MB functionalization since click reaction is highly efficient to incorporate a broad set of additives (nanoparticles, siRNA, antibodies).

Therefore, the chemistry behind MB functionalization involves a predefined set of strategies to implement. Furthermore, the presence of amino acids with different radicals in the structure of protein MBs allows for quick incorporation of the necessary functional fragments into MB structure. Hence, protein MBs can act as efficient agents for multimodal imaging and image-guided therapy applications.

## 5. Advanced Characterization of MBs with a Protein Shell

**Figure 5:**
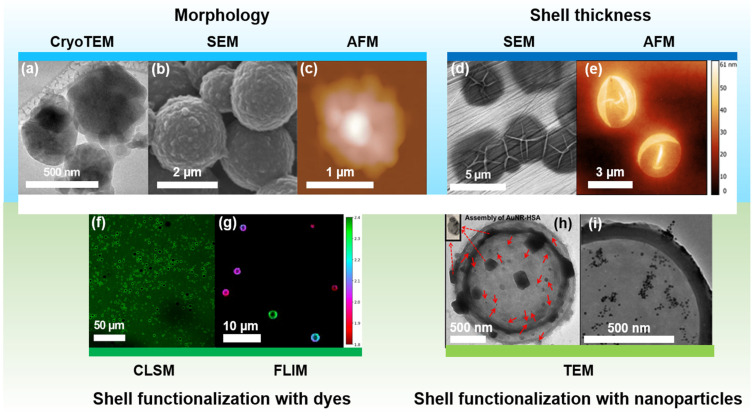
Characterization of MBs with a protein shell: methods to evaluate morphology, shell thickness, and functionalization of fabricated agents. Morphology evaluation: (**a**) CryoTEM image of BSA MBs; (**b**) SEM image of modified lysozyme MBs. Adapted with permission from Ref. [89]. Copyright 2013, American Chemical Society; (**c**) AFM image of lysozyme MBs. Adapted with permission from Ref. [133]. Copyright 2008, American Chemical Society; Shell thickness measurements: (**d**) SEM and (**e**) AFM images of BSA MBs were used to evaluate shell thickness; confirmation of shell functionalization with dyes: (**f**) CLSM and (**g**) FLIM images of rhodamine-labeled BSA MBs; Confirmation of shell functionalization with nanoparticles: (**h**) TEM image of gold nanorods (AuNRs) functionalized HSA MBs. Adapted with permission from Ref. [145]. Copyright 2012, SPIE; (**i**) TEM image of gold nanoparticles (AuNPs) functionalized lysozyme MBs. Adapted with permission from Ref. [89]. Copyright 2013, American Chemical Society. Abbreviations: cryoTEM, transition electron cryomicroscopy; SEM, scanning electron microscopy; AFM, atomic force microscopy; CLSM, confocal laser-scanning microscopy; FLIM, fluorescence lifetime imaging microscopy; TEM, transition electron microscopy. Where red arrows indicate single AuNRs and dashed ones point to assemblies of AuNR and HSA.

The expedient choice of characterization method is essential to fully evaluate the properties of protein MBs, as recently demonstrated for dye-labeled BSA MBs [138]. In this section, we summarize existing approaches in MB characterization, providing examples in Figure 5.

After MB fabrication, size distribution and concentration properties must be evaluated first. MB size can directly affect their US response, while high and monodisperse MB yield raises the reasonability of agent production [139,140]. Few measurement approaches could be considered. The traditional one is based on combining optical microscopy (OM) with a cell counter, where MBs are counted manually in a time-consuming manner [19,21,90,138,141,142]. Another approach involves Coulter counter (CC) measurements, based on resistive pulse sensing for counting and sizing particles suspended in electrolytes. The CC device reduces the quantification time significantly [20]. In contrast, two light scattering-based methods, dynamic light scattering (DLS) and nanoparticle tracking analysis (NTA), can be considered. However, both methods result in distorted size distribution due to initial high MB size polydispersity. Moreover, it should be noted that the accuracy of measurements is strongly dependent on sample dilution [143,144]. Therefore, CC measurements are more favorable than other methods in terms of ease of operation and reliability of obtained results.

For characterization of MB morphology, transmission electron cryomicroscopy (TEM), scanning electron microscopy (SEM), and atomic force microscopy (AFM) methods were successfully implemented, examples are provided in Figure 5a–c, respectively [94,133,145,146]. SEM of broken MBs and AFM of dried MBs can provide precise information about MB shell thickness, as illustrated in Figure 5d,e, respectively [90,146]. AFM of liquid samples can shed light on MB shell stiffness profile, acting as the only method for Young modulus evaluation of MB shell [146]. However, only a few articles considered AFM for MB characterization due to the MB fragility and sophisticated sample preparation route.

The introduction of fluorescent small molecules can be confirmed by confocal microscopy (Figure 5f) [84,109,133,143,147], while STED or fluorescent lifetime imaging microscopy (FLIM) may offer higher image resolution combined with insight into the homogeneous/heterogeneous distribution of fluorescent components in the shell and distribution of dye among MB population (Figure 5g) [148]. Unfortunately, FLIM and STED methods are barely described for protein MBs [138]. The presence of inorganic nanoparticles can be confirmed with TEM (Figure 5h,i) [89,145]. MB drug loading capabilities are usually evaluated with spectrophotometry or chromatography [20].

The storage stability of protein MBs can be evaluated by zeta-potential measurements, in addition to methods described for size/concentration characterization [19,70,109,138,149]. However, significant attention should be given to the stability of MBs in blood-mimicking solutions, resulting in the evaluation of a parameter similar to in vivo circulation time. It can be done with vessel-mimicking phantoms with predefined pumping speed. Similar phantoms are generally constructed to evaluate the acoustic response of fabricated MBs.

Therefore, a straightforward strategy to assess fluorescent drug-loaded protein MBs (i.e., doxorubicin-loaded HSA MBs, as clinically-relevant anticancer formulation) could be: (1) to characterize the size and concentration via CC, (2) to assess morphology and drug loading using STED or FLIM and spectrophotometry, (3) to evaluate stability and acoustic response in vessel mimicking phantom and the solution mimicking blood composition.

## 6. Applications of MBs with Protein Shell beyond US Imaging

**Figure 6:**
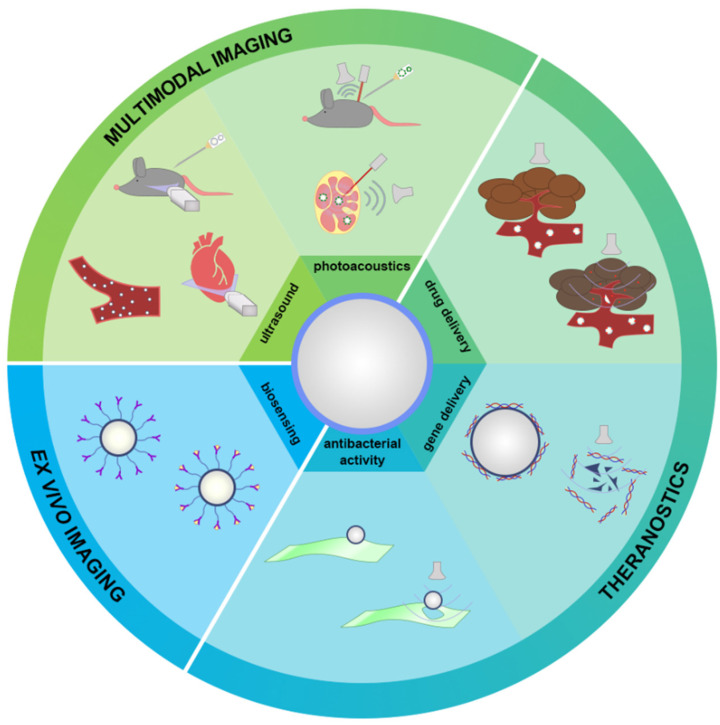
Applications of MBs with protein shell. Historically, protein MBs were the first contrast agent for cardiac US imaging, while recent advances in photoacoustic imaging expanded their multimodal imaging applications. Theranostic strategies result in US-assisted drug and gene delivery and antibacterial purposes based on US-assisted MB destruction. Ex vivo imaging purposes demonstrated with MB-assisted biosensing can widen the range of MB applications.

Historically, MBs entered clinical practice as US contrast agents. Over the past 20 years, the research interest in MBs shifted from US imaging to combined image-guided/multifunctional strategies. In addition to US imaging, MBs gained applications in photoacoustic (PA) imaging, targeted and image-guided drug or gene delivery, antibacterial activity, and biosensing, as schematically represented in Figure 6. This section provides all known options for multifunctional applications of protein-based MBs.

### 6.1. Imaging Applications

PA and US are complementary imaging methods since both strategies receive acoustic echo to construct resulting images. In addition, absorbers, such as organic dyes or plasmonic nanoparticles, can tune the resulting PA images (as MBs for US imaging). The introduction of dyes into the MB structure has been known for a long time [72]. Recently, the introduction of PA dyes, such as methylene blue [96] or indocyanine green [19], has been demonstrated. Furthermore, it has been shown that US and PA signals can be controlled independently by changing the dye concentration in the MB structure, which was confirmed by in vivo studies. Such examples may represent promising bimodal agents for brain imaging or sentinel lymph node detection (Figure 6) [150,151,152,153,154].

Beyond US cardiac imaging, protein-shelled MBs can be used for imaging the endothelium [72], vessels with atherosclerotic plaques [110,111], and detection of inflammatory foci (Figure 6) [69,72]. It has been shown that MBs do not adhere to normal human endothelial cells but can adhere to inflamed lesions. We may name several reasons for this effect. Endothelial cell surface proteins, such as leukocyte adhesion molecules, are activated and expressed during inflammation [155,156,157]. Hence, they may have an affinity for protein MBs through protein-protein interaction. Furthermore, the inflammatory response includes the synthesis of new matrix components and the degradation of pre-existing ones [158]. Thus, matrix degradation products also may show an affinity for MBs. It is also possible that albumin in the bladder membrane is involved in adhesion because albumin binds in vivo to the endothelial glycocalyx via at least four putative albumin-binding proteins [159,160]. Moreover, endothelial damage can occur due to a variety of causes: arterial hypertension, hyperlipidemia, diabetes mellitus, coronary angioplasty, or postischemic reperfusion, and result in atherosclerosis, thrombosis, or restenosis [161,162,163]. Early atherosclerotic lesions and predisposition to thrombosis coincide with endothelial cell protein activation and leukocyte adhesion molecules activation [161,164,165]. Thus, MBs with the protein shell have an advantage for early detection of inflammation due to the mentioned reasons. MB-endothelial adhesion could eventually be extended to developing contrast agents that target specific markers of the cellular phenotype, opening up opportunities for tissue-specific contrast US imaging.

### 6.2. Drug/Gene Delivery

Protein MBs are widely involved in cancer treatment strategies. In [71], albumin MBs with gold nanoparticles (as plasmonic nanoparticles) and VEGFR2 antibodies (as targeting ligands) adsorbed on the surface were used for targeted photothermal therapy. After binding to angiogenesis markers, MBs were sonicated to release the therapeutic agent confirmed by PA measurements. Yoon et al. [137] also used gold nanoparticles prone to aggregation: after US-mediated MB destruction, nanoparticles entered tumor lesions via sonoporation and aggregated. Then, photothermal therapy was applied. In addition, photodynamic therapy can be optimized with MBs since it is relevant to deliver not only photodynamic agents but also oxygen (since hypoxic conditions occur in tumors typically), while photodynamic agents can be delivered to the tumor site with sonoporation [166,167,168,169]. Previously, photodynamic dyes (indocyanine green, zinc phthalocyanine) and gold nanoparticles were implemented on the albumin MB shell [19,21]. Narihita et al. [68] developed albumin MBs coated with cetuximab for theranostics of oral squamous cell carcinoma (HSC-2). The cell killing rate during sonication in the presence of cetuximab was higher than for non-targeted albumin MBs. On the other hand, selective cell killing was not observed in the human myelomonocytic lymphoma line (U937) with no cetuximab affinity. Another anticancer drug, doxorubicin, was used for protein MB formulation in [104,170,171]. Due to hydrophobic interactions, drug molecules were sorbed on lysozyme MBs, and MBs showed promising treatment results. Thus, protein-based MBs can potentially be used for theranostics as drug delivery vehicles, enhancing therapeutic effects in cancer treatment (Figure 6).

Several studies have shown that albumin MBs can effectively bind nucleic acids and synthetic oligonucleotides (Figure 6) [172]. MBs can directly capture genetic material such as plasmids and adenoviruses. The first published report on targeted DNA delivery was performed in 1996 using intravenously delivered MBs containing oligonucleotides [53]. In 1997, Bao et al. [173] described the use of US and albumin-coated MBs to enhance transfection of the luciferase reporter plasmid in cultured hamster cells. Shohet et al. [174] demonstrated that US-mediated destruction of gas-filled MBs can be used for direct gene expression to the heart in vivo. Intravenously administered recombinant adenoviral vectors encoding the beta-galactosidase reporter gene were successfully delivered to normal rat myocardium using MBs and a 1.3 MHz transthoracic diagnostic US device with a mechanical index of 1.5. Of note, no transfection was observed if adenovirus was administered at the same dose without MBs or if adenovirus was administered with MBs, but US was not applied [175]. Nowadays, many studies have confirmed the effectiveness of US-mediated MB destruction for both in vitro and in vivo drug and gene delivery [80,81,109,136,174,176,177].

### 6.3. Antibacterial Activity

In 1922, before discovering penicillin, Alexander Fleming discovered that lysozyme inhibits bacterial growth [178]. Lysozyme is a natural enzyme found in body secretions such as tears, saliva, and milk and is considered part of the innate immune system of most mammals [179]. Lysozyme destroys peptidoglycan in the bacterial cell wall, leading to cell death [180]. Lysozyme was already used to form MBs [93]. Lysozyme MBs can also partially retain their antimicrobial activity despite changing protein conformation (Figure 6) [133].

Hence, Mahalingham et al. [87,121] demonstrated the antibacterial activity of lysozyme-based microbubbles against Gram-negative *Escherichia coli* (*E. coli*). Another study [88] investigated a novel strategy for acne treatment based on the antibacterial action of lysozyme MBs and US-mediated cavitation both in vitro and in vivo, aiming to reduce the dose and duration of treatment. As a result, the growth of *P. acnes* bacteria was inhibited by 86.08 ± 2.99%. Furthermore, MBs can have not only antibacterial but also antimicrobial activity [89]: the introduction of gold nanoparticles in the MB structure can exhibit antimicrobial activity and demonstrate effectiveness against *M. lysodeikticus.*

### 6.4. Biosensing

Biosensing is an intriguing MB application beyond in vivo imaging and therapy (Figure 6). While many cell sorting techniques were already described, including fluorescence-activated or magnetic field-activated ones, mechanical forces can damage cells during a procedure. Liou et al. [82] demonstrated the MB-assisted method, buoyancy-activated cell sorting, which involves MBs composed of biotinylated albumin conjugated to anti-CD44 antibodies. MBs were implemented to isolate breast cancer cells, and over 90% of the cells were collected in the microbubble layer. CD44+ is a widely used cancer stem cell biomarker. Thus, the described agents could be a powerful tool for sorting cancer stem cells from dissected tumor tissue. Another option of MBs used as biosensors was described in [89]. The interface of the lysozyme-based MBs was modified with alkaline phosphatase to detect the presence of paraoxon in aqueous solutions at the lowest concentrations down to 1 ppm.

Thus, the range of applications of protein-based MBs covers not only US imaging but also other imaging and therapeutic strategies and biosensing options. These strategies directly depend on the MB structure: gaseous core, a primary protein used for shell stabilization, and functional additives.

## 7. Conclusions

Protein MBs are still clinically available US contrast agents with the example of Optison with the HSA-stabilized shell. HSA is considered the most well-discovered protein with clear perspectives for clinical translation of HSA-based solutions. Hence, HSA-stabilized MBs can be produced with a concentration up to 10^10^ MBs/mL, tuned mean diameter in the range of 1–7 μm, and circulation time of 1–2 min [20,48,49,53,54,55]. However, the introduction of stabilizing agents in the BSA shell of agents can improve properties of low stability and short circulation time [80,109]. Moreover, large-scale production of monodispersed HSA MBs, a few microns in mean diameter, remains a point for improvement [79,128,129]. Beyond albumin, oleosin (recently involved in microfluidic narrow-sized MB fabrication) [95,96] and lysozyme (with advantages in antibacterial activity and biosensing) [87,89,121] are the most relevant proteins for MB production.

Therefore, MBs with the protein shell demonstrates a predefined set of proteins used for shell stabilization, as well as fabrication routes aimed at large-scale production; well-discovered functionalization routes (non-covalent as electrostatic, hydrophobic, “key-lock” interactions or covalent, by carbodiimide synthesis or with the introduction of additional crosslinking reagents) [80,82,134], and a broad set of biomedical applications (anticancer therapy, drug/gene delivery, antibacterial activity, and biosensing) [19,71,87,89,96,121]. Hence, protein MBs are superior platforms for the practical translation of smart agents.

Further research should be considered on the topics of:

(1) Systematic evaluation of strategies to prolong protein MB stability (both storage stability and stability during imaging/treatment procedure) with the precise attention to biocompatible additives that can be easily incorporated into MB shell and can shift MB shell properties to hard as polymer MBs;

(2) Large-scale production of functional MBs with narrow size distribution and high production yield involving predefined routes for MB functionalization directly during MB fabrication;

(3) Critical evaluation of MB behavior under conditions close to natural for a better understanding of their efficiency in practical clinical applications in imaging and therapy and reasonable design of smart agents;

(4) Further exploration of MB applications for biological barriers opens research on brain disorders, transdermal drug delivery, and smart anticancer therapy [170,181,182,183,184,185,186].

US-sensitive stimuli-responsive MBs open the door for advanced cancer treatment and biosensing procedures based on the effects of US-mediated MB destruction, sonoporation, and sonopermeation. Thus, combining US-guided strategies and protein MB advantages (mostly by protein-protein interactions) can lead to optimal procedures relevant for clinical practice in the short term.

## Figures and Tables

**Table 1:** Fabrication of MBs with protein shell by microfluidics. All examples within each type of microfluidic device are provided in chronological order.

Microfluidics Type	Primary Shell Material	Additives	Gaseous Core	Size (µm)	Ref.
Flow-focusing	BSA (3%, 5%)	None/ Dextrose/Glycerol, propylene glycol, and isotonic saline	N_2_	10–20	[124]
	Oleosin	Pluronic F68/Pluronic F127	N_2_, C_4_F_8_	3.9 ± 0.2	[125]
	BSA (3%)	Dextrose (10%)	N_2_	9.1–19.8	[126]
	BSA (4%)	Dextrose (10%)	N_2_	9.8 ± 0.3–31.1 ± 1.4	[127]
	Oleosin	Pluronic F68/Pluronic F77/ Pluronic F105/ Pluronic P65	N_2_	2–4	[95]
	Oleosin	Pluronic F68, Methylene Blue	N_2_	2–4	[96]
T-junction	BSA (15%)	None/ Tween 40/phospholipid solution	Air	81 ± 2–555 ± 3	[128]
	BSA (15%)	-	N_2_	272 ± 5	[129]
	BSA (15%)	None/Glutaraldehyde (0.75%)	N_2_	270 ± 2	[79]

## Data Availability

No new data were created or analyzed in this study. Data sharing is not applicable to this article.

## Abbreviations

AFM, atomic force microscopy; BHIA, baffled high-intensity agitation; BSA, bovine serum albumin; CC, Coulter counter; CEHDA, coaxial electrohydrodynamic atomization; CEUS, contrast-enhanced ultrasound; CLSM, confocal laser-scanning microscopy; cryoTEM, transition electron cryomicroscopy; DLS, dynamic light scattering; FDA, the Food and Drug Administration; FLIM, fluorescence lifetime imaging microscopy; HSA, human serum albumin; MBs, microbubbles; NTA, nanoparticle tracking analysis; OM, optical microscopy; PA, photoacoustic; SEM, scanning electron microscopy; STED, stimulated emission depletion microscopy; TEM, transition electron microscopy; US, ultrasound.
